# Reduction in IUSS (Immediate Use Steam Sterilization) Associated with Reduction in Surgical Site Infections

**DOI:** 10.1017/ash.2024.226

**Published:** 2024-09-16

**Authors:** Jennifer Jaffe, Linda Merz, Kathleen McMullen

**Affiliations:** Mercy; Mercy Hospital South

## Abstract

**Background:** Immediate use steam sterilization (IUSS) is a potential risk factor for surgical site infection (SSI). During a regulatory survey, it was discovered that IUSS rates for a 767-bed hospital exceeded what had been reported to Infection Prevention (IP) and surgery leaders (estimated at an average of 60 instances per month, with approximately 40 of those in orthopedic cases). A Quality Improvement (QI) project to reduce IUSS was implemented. **Methods:** The QI project started with the requirement of three signatures for every cycle of IUSS (surgery management, sterilization management, and IP). Additional trays were ordered to provide an ample supply for cases. Surgery personnel were no longer allowed to perform IUSS, and the number of sterilizers available for IUSS was reduced from 8 to 1. The project was fully implemented as of December 2019. To evaluate the impact, SSI rates for hip and knee prothesis were compared using chi square analysis (Epi Info, CDC); before QI project rates were measured from 2017-2019 and after QI project rates were measured for 2020-2022. No other changes were made that were anticipated to impact orthopedic SSI rates. **Results:** There were no instruments or implants processed by IUSS after December 2019. Prior to the project, there were 9 hip SSI (rate = 0.54 per 100 procedures) and 14 knee SSI (rate = 0.49 per 100 procedures). After the project, hip SSI decreased by 76% (2 SSI, rate = 0.13 per 100 procedures, p = < 0 .05) and knee SSI decreased by 18% (7 SSI, rate = 0.41 per 100 procedures, p=0.67). **Conclusion:** A multidisciplinary QI project was successful at drastically reducing the use of IUSS, and a correlating statistically significant decrease in hip SSI and clinically significant decrease in knee SSI was seen for 3 years after the project was completed.

**Disclosure:** Kathleen McMullen: Speaker - 3M

